# Food Retail Resilience Pre-, during, and Post-COVID-19: A Bibliometric Analysis and Research Agenda

**DOI:** 10.3390/foods13020257

**Published:** 2024-01-13

**Authors:** Rebeka-Anna Pop, Dan-Cristian Dabija, Cristina Bianca Pocol

**Affiliations:** 1 Department of Marketing, Faculty of Economics and Business Administration, Babeș-Bolyai University, 400591 Cluj-Napoca, Romania; rebeka.pop@ubbcluj.ro; 2 Department of Animal Production and Food Safety, Faculty of Veterinary Medicine, University of Agricultural Sciences and Veterinary Medicine, 400372 Cluj-Napoca, Romania

**Keywords:** COVID-19, food retail, strategy, resilience, bibliometric analysis, B2B, B2C

## Abstract

This paper aims to conduct a bibliometric mapping and systematic review of the food retail industry’s resilience strategy in the context of COVID-19. Specifically, we aim to identify relevant research gaps in the industry during the pre-, during, and post-pandemic periods and highlight the differences between B2B and B2C sectors. We analyzed articles in the Scopus database from 2019 to 2022 using the PRISMA method for article selection, resulting in a total of 69 articles. We employed a VOS viewer for bibliometric mapping. Our analysis revealed that most studies focused on the impact of COVID-19, with only a few examining the pre- and post-pandemic periods critically. In the B2C context, we identified two major topics: changes in purchasing and consumption behavior, and food waste and safety. In the B2B sector, the two most recurrent subjects were retailers’ strategies and supply chain management. This study provides valuable insights for policymakers by exploring industry trends and for scholars by highlighting future research agendas based on the identified topics.

## 1. Introduction

The COVID-19 pandemic has significantly impacted the food retail industry worldwide. The pandemic has not only led to a shift in consumer shopping and consumption behavior, but it has also brought about significant changes in the supply chain system of the food retail industry. As a result, researchers and scholars have turned their attention to the study of food retail resilience in the pre-, during, and post-COVID-19 periods. This bibliometric analysis aims to identify and synthesize the existing body of knowledge in this area, and to propose future research agendas.

The COVID-19 pandemic has caused significant disruptions in the food retail industry, affecting both the companies and the consumers. Consumers have been forced to change their shopping and consumption behavior due to lockdowns, social distancing guidelines, and the fear of infection. This has led to a surge in online shopping and a shift towards home cooking, with consumers increasingly looking for healthy and immune-boosting food products [1]. On the other hand, companies have been facing various challenges such as supply chain disruptions, labor shortages, and declining sales [2]. In response to the challenges posed by the pandemic, companies have implemented various strategies to ensure business continuity and resilience. These include the adoption of e-commerce and online ordering platforms, the implementation of contactless delivery and pickup options, and the introduction of safety measures for both employees and customers. These strategies have enabled companies to adapt to the changing business environment and to continue to provide essential food products to their customers.

In the academic field, many scholars have explored the impact of the COVID-19 pandemic on the food retail industry. The research has focused on various aspects such as the changes in consumer behavior and preferences, the supply chain disruptions and resilience, and the impact on the food retail businesses. As a result, a significant number of articles have been published in academic journals since 2020. The objective of this study is to synthesize the existing body of knowledge in the context of food retail resilience in the pre-, during, and post-COVID-19 periods. To achieve this, we employed a bibliometric analysis to map the existing trends in this area. The analysis was based on a sample of 69 articles, which were analyzed to identify specific trends in publishing journals, used keywords, and citation network. Furthermore, a systematic literature review process was employed to explore in-depth the results of the selected studies. This enabled the authors to draw relevant conclusions and discuss future research agendas. The study provides valuable insights into the pre-, during, and post-COVID-19 trends in the food retail industry. It also formulates trends and future research agendas for both B2B and B2C sectors, considering the impact of the pandemic on companies and consumers alike. The results of this study are expected to be of significant value to researchers, policymakers, and practitioners in the food retail industry.

Our study is organized as follows: Section 2 represents the methodology process of the bibliometric analysis; Section 3 covers the results of the systematic mapping. In Section 4, we discuss the findings of the study, focusing on two major topics: the impact of COVID-19 on the food retail industry in the pre-, during, and post-pandemic periods, and the differences between B2B and B2C sectors. Section 5 includes a set of recommendations and future research agenda on the topic, based on the identified research gaps and emerging trends, and Section 6 explains the theoretical and managerial implications, together with the limitations and future research perspectives.

## 2. Materials and Methods

### 2.1. Source of Information

Accurate and reliable bibliographic data is essential for research across different fields. The study obtained such data from Scopus, which is a database created by Elsevier in 2004. Scopus contains a vast collection of research output from over 7000 international publishers, totaling more than 87 million documents [3]. This database offers a range of information, including author profiles, citation data, and article metrics, making it a valuable tool for researchers who want to evaluate their work’s impact or identify potential collaborators. Additionally, Scopus offers advanced search features and filters, enabling authors to perform precise and detailed searches, and ensuring quick and efficient retrieval of accurate and relevant results [3].

### 2.2. Search Strategy

Bibliometric analysis is a critical tool for conducting research in various fields, providing researchers with valuable insights into the publication patterns, research trends, and collaborations among researchers in a particular field [4,5]. The following paragraphs describe the steps taken in the bibliometric analysis conducted in this study, focusing on the selection of appropriate keywords, search strategy, and data collection process. In this regard, the bibliometric analysis in this study follows the recommended steps by Sinkovics [6] and begins with defining the search strategy and data collection. The selection of appropriate keywords is critical for effectively searching, accessing, and interpreting relevant data in the field. The study focuses on analyzing the resilient retail strategy before, during, and after the COVID-19 pandemic, and therefore the study period was set from 2019 to 2022, aligning with the outbreak and end of the pandemic. To optimize the search strategy, a combination of Boolean operators OR and AND was used to combine the selected keywords, including ‘retail resilience strategy’, ‘COVID-19’, ‘coronavirus’, and ‘SARS-CoV-2’. These keywords were selected to focus on the resilient retail strategy during the COVID-19 pandemic (Table 1). The study specifically selected research articles and review articles from the fields of Business, Management, and Social Sciences that were written in English. Initially, we conducted a broad search using the above-mentioned keywords and found a substantial number of articles published with topics related to ‘COVID-19’, ‘Coronavirus’, or ‘resilience’. As a result, we realized that a more precise approach was necessary to accurately identify the sample database. To achieve this goal, we followed the PRISMA guidelines introduced by Moher et al. [7]. The PRISMA guidelines are designed to ensure transparency and rigor in the selection of studies for a systematic review or meta-analysis. The four distinct steps include identifying the research question, selecting relevant studies, extracting data from the selected studies, and assessing the quality of the studies.

**Figure 1:**
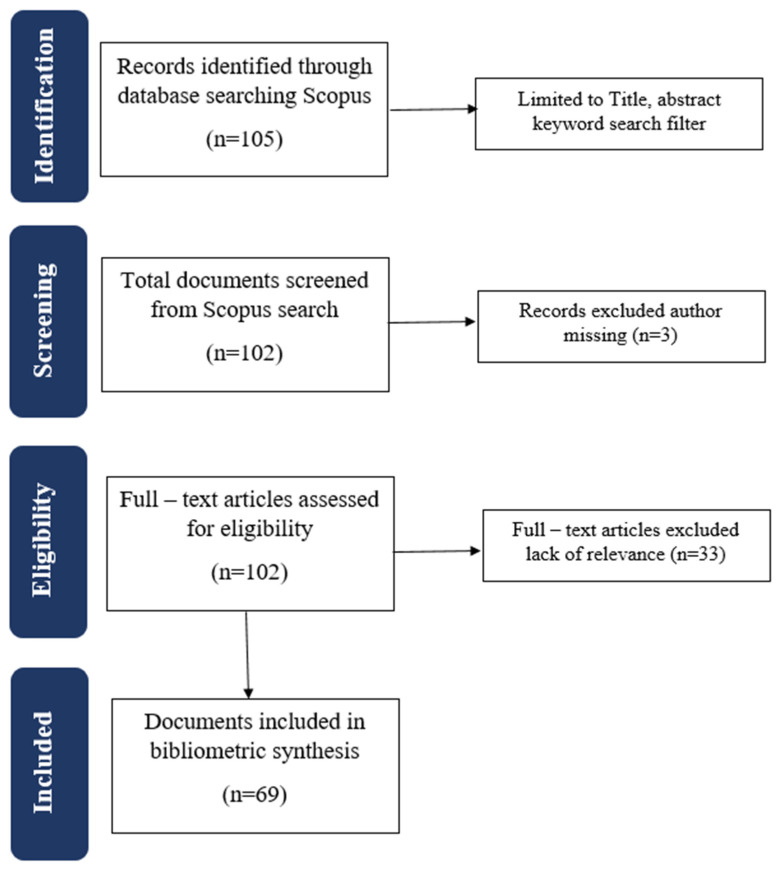
The PRISMA flow diagram is used to identify, screen, and include papers.

To adhere to the PRISMA guidelines, the first step of this study involved identifying relevant articles on the subject of resilient retail strategy during the COVID-19 pandemic (Figure 1). To manage the vast amount of data available, the keyword search was restricted to the title, abstract, and keywords to ensure the articles’ relevance, resulting in 105 articles. The screening process continued, and in the next step, we eliminated articles without author names, resulting in 102 articles. Fortunately, we did not find any duplicates during this stage. The next step involved qualitative synthesis, where each article was carefully analyzed by reading its title and abstract and assessing whether it corresponded to the research’s purpose. For this, we used the Covidence platform in order to organize the systematic review process. During this process, 33 articles were removed from the final analysis due to irrelevant topics poor-quality methodology, or interpretation of results, leaving a total of 69 articles for the bibliometric analysis.

The bibliometric analysis encompasses various analytical elements, such as total citation counts, geographical distribution, institutional affiliations, sources, and author-developed keywords. This approach provides a comprehensive literature analysis, allowing for a detailed examination of the bibliographic data [8,9]. VOS Viewer 1.6.19 software enables the identification of patterns and trends within the literature enhancing the understanding of the theoretical foundation of resilient retail strategies before, during, and after COVID-19.

## 3. Results

**Figure 2:**
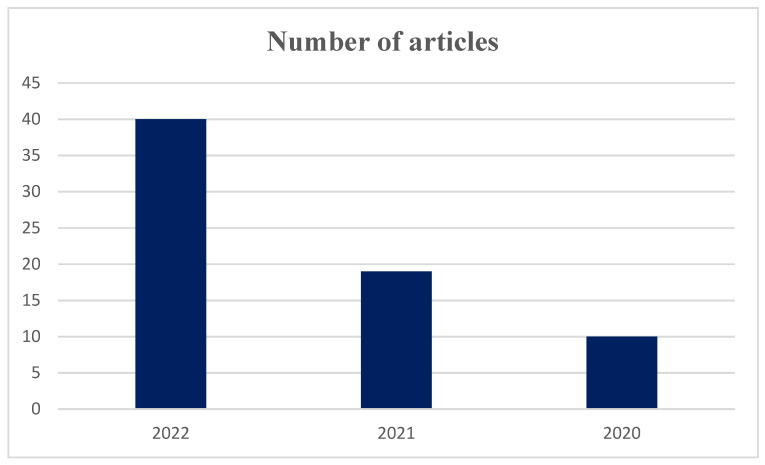
Quantifying Articles on Retail Resilience in the Context of COVID-19.

The findings indicate that the quantity of publications addressing retail resilience during the COVID-19 pandemic has shown a consistent upward trend over the last three years (Figure 2). Specifically, in 2020, which marked the initial year of the pandemic, there were 10 articles published on this subject. This number nearly doubled in 2021, with 19 articles published. The trend continued in 2022, with a significant increase in the number of articles, reaching 41 in total. This observation suggests that the academic community is increasingly recognizing the importance of studying the resilience of the retail sector in response to the pandemic, as well as the need to explore effective strategies for enhancing resilience. Moreover, the publication trend also highlights the dynamic and evolving nature of the pandemic’s impact on the retail sector. The first year of the pandemic was characterized by rapid changes and challenges, with retailers facing significant disruptions to their operations and supply chains. As the pandemic continued into 2021, retailers adapted to the ‘New normal’, and research focused on evaluating the effectiveness of different resilience strategies. By 2022, with the vaccine rollout and lifting of restrictions in some regions, researchers shifted their attention to examining the lasting effects of the pandemic on the retail sector and the long-term resilience strategies that can help businesses survive and thrive in the post-pandemic world.

Moreover, Table 2 provides an overview of the journals in which the articles included in the analysis were published. It is noteworthy that a significant number of articles were concentrated in two prominent journals, namely ‘Sustainability’, which had ten articles, and ‘Socio-Economic Planning Sciences’, which had five articles. Additionally, journals such as ‘Food Security’ and ‘Global Food Security’ each had three published articles related to the topic. What makes this particularly intriguing is that several journals that are not typically associated with consumer studies or marketing have also made significant contributions to the literature on this subject. This is due to the multidisciplinary nature of consumer studies, as well as the unique context of the COVID-19 pandemic’s impact on the food retail sector. As the pandemic continues to evolve, researchers must adopt a multidisciplinary approach to studying the resilience of the retail sector. The inclusion of journals from various fields reflects a growing recognition of the need for a holistic understanding of the challenges and opportunities facing the food retail industry during the pandemic.

### 3.1. Bibliometric Analysis of the Document Citation

**Figure 3:**
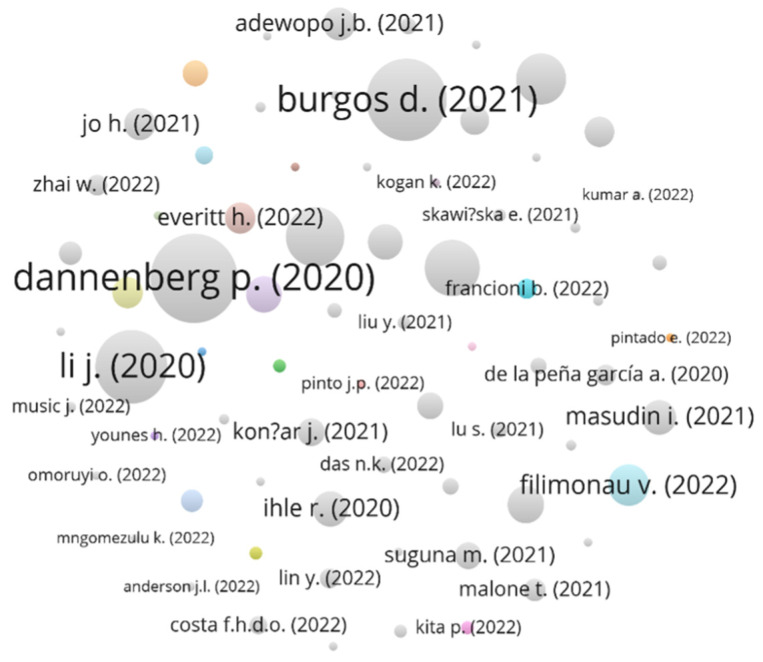
Co-citation of cited references.

The purpose of this analysis is to investigate the interrelationships among documents in the SCOPUS database based on the number of citations they received. Reciprocal citation links between articles are used to indicate connections between them, as depicted in Figure 3.

Of all the articles analyzed, ‘Digital Transition by COVID-19 Pandemic? The German Food Online Retail’ by Dannenberg [10] has received the highest number of citations, with a total of 138 according to SCOPUS data, yet no reciprocal links were found. Following closely behind is the article by Burgos and Ivanov [11], entitled ‘Food retail supply chain resilience and the COVID-19 pandemic: A digital twin-based impact analysis and improvement directions’, which has been cited 118 times without any reciprocal links. The article by Li et al. [12] on ‘Changing Grocery Shopping Behaviours among Chinese Consumers at the Outset of the COVID-19 Outbreak’ has also received significant attention, with 94 citations and no reciprocal links. Interestingly, although these highly cited articles were published around the same time, they do not have reciprocal links to each other.

This observation suggests that they were independently identified as crucial contributions to the literature on COVID-19 and its impact on the food retail industry. However, future research may uncover new connections and relationships between these highly cited articles, providing further insights into the evolving nature of research in this field. As research on the COVID-19 pandemic and its impact on the food retail sector continues to grow, it is crucial to examine and understand the interconnections among different studies. Such analysis can help identify important research gaps and areas for future exploration, as well as highlight the interdisciplinary nature of research in this field.

### 3.2. Bibliometric Analysis of the Document Citation

**Figure 4:**
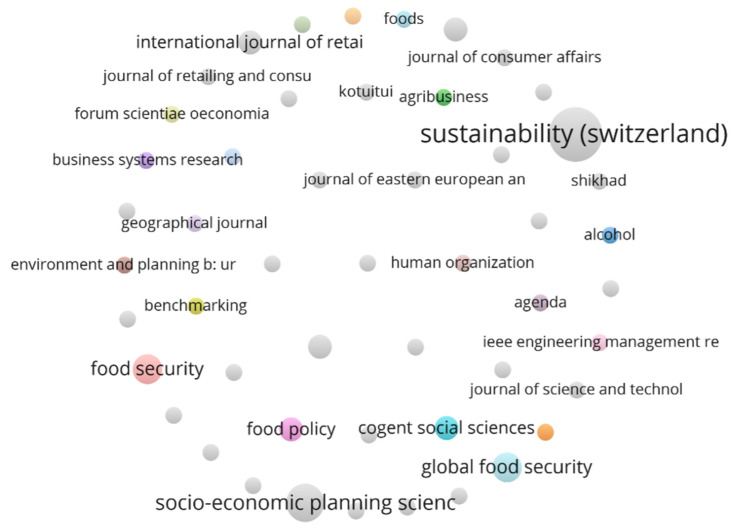
Journal co-citation network.

This analysis presents a co-citation study that investigates the citation network of journals, aiming to understand the relationships among them. In this study, circle size is used to denote the number of published works, while the links between circles represent the frequency of citations (Figure 4). Shorter distances between journals indicate higher citation frequency, implying stronger connections between them. Initially, out of 50 sources, only four journals met the minimum citation threshold of three for being included as a cited source in the analysis. However, due to the limited number of journals meeting this threshold, the threshold was lowered to one, resulting in a total of 50 journals being included in the analysis. This allowed for a broader exploration of the citation network among journals.

The results of the analysis revealed that the Sustainability journal had a significant presence in this field, with 10 published articles, making it the top contributor. However, it did not show any links to other journals in the co-citation network, which suggests that it may be operating independently or not strongly connected to other journals in terms of citation relationships. In contrast, the Socio-economic planning sciences had a moderate presence in the citation network, with a total of 5 documents published on the topic, but it also did not show any links to other journals in the co-citation network. One possible explanation for the lack of interconnections among journals in the co-citation network could be the novelty of the topic. If the topic is relatively new or emerging, it may take time for the journals to establish strong citation relationships with each other. Additionally, it could be due to differences in research focus, methodology, or scope among the journals, resulting in limited cross-references in their publications. However, as more research is conducted in this field, the connections between journals will likely become stronger and more pronounced.

### 3.3. Country Co-Author Analysis

**Figure 5:**
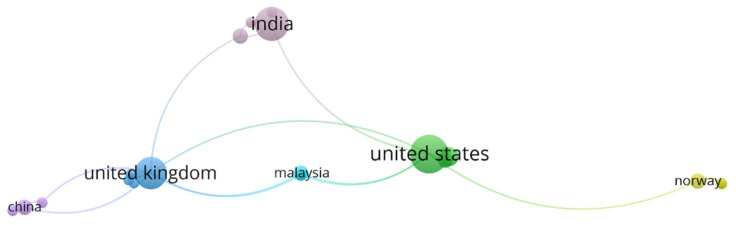
Countries’ co-authorship network.

Furthermore, the co-authorship analysis provides valuable insights into the research collaboration networks in the field of resilient strategies in the context of COVID-19 in the retail sector (Figure 5). The prominence of the USA, the United Kingdom, and India as leading nations in terms of the number of documents published on this topic is noteworthy, with 13, 9, and 10 documents respectively. The analysis also indicates that there is some level of collaboration between these nations, as evidenced by the connections between their nodes in the network. This suggests that research on retail resilient strategies in the context of COVID-19 is a globally collaborative effort involving researchers from multiple countries.

### 3.4. Bibliometric Analysis of the Keywords

**Figure 6:**
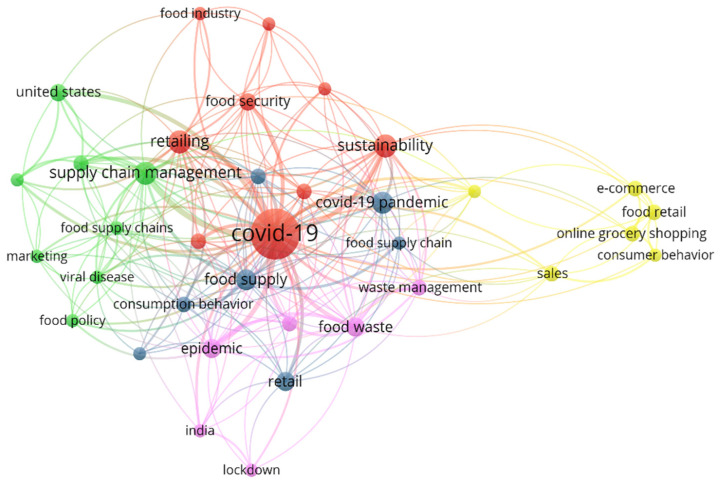
Co-occurrence network of articles based on keywords.

The findings from the analysis of keyword occurrences using VOS viewer reveal interesting insights. Despite the default minimum threshold for keyword occurrences being set at five in the VOS software 1.6.19, we lowered it to three due to the small number of articles included in this study. As a result, 36 keywords met the lowered threshold out of the initial 449 keywords identified, indicating the relevance of a wide range of concepts in the literature on retail resilience in the context of COVID-19. Among the keywords with the highest link strengths, ‘COVID-19’ and ‘Supply chain management’ stand out, with total link strengths of 158 and 52, and with a total number of occurrences of 42 and 9, respectively. This suggests that these two concepts are central to the research on retail resilience during the pandemic. Additionally, keywords such as ‘Retailing’ and ‘Food supply’ also emerged as frequently used keywords, each with a total link strength of 50 and 42, indicating their significance in the literature. The visualization generated using VOS viewer (Figure 6), where keywords or concepts are represented as nodes with their size corresponding to their frequency, following the methodology proposed by van Eck and Waltman [13], provides a clear visual representation of the prominence of different keywords in the literature. The use of different colors to represent each cluster of keywords or concepts further aids in identifying distinct patterns or themes, resulting in the identification of six distinct clusters in the analysis. This indicates the multidimensionality and complexity of the topic, with different clusters representing different aspects of retail resilience in the context of COVID-19.

Cluster one, shown in red, is centered around ‘COVID-19’ and includes related keywords such as ‘sustainability’, ‘food security’, and ‘food industry’. This cluster specifically pertains to the impact of the pandemic on retail businesses. Cluster two, depicted in green, prominently features ‘Supply chain management’ as a node and groups together keywords such as ‘price dynamics’ and ‘marketing’. This cluster primarily focuses on the strategies employed by retailers, particularly in the context of food retail and resilience. Cluster three, represented by the blue color, is centered around ‘food supply’ and ‘consumption behavior’. This cluster serves as a combination of the other clusters, potentially representing common themes or overlaps among the keywords from different clusters. Cluster four, shown in yellow, has ‘sales’ as a prominent node and includes keywords such as ‘consumer behavior’, ‘e-commerce’, and ‘online grocery shopping’. This cluster reflects consumer behavior and decision-making processes, particularly in the context of online shopping. Cluster five, depicted in purple, ‘epidemic’ as a prominent node and groups together keywords such as ‘food consumption’ and ‘food waste’. This cluster also relates to the pandemic, but differs from Cluster 1 in terms of context, as it focuses on the impact on food consumption and waste, rather than business strategies. Despite the presence of similarities in keywords across clusters, each cluster emphasizes a distinct aspect of retail resilience in the context of COVID-19, highlighting the multidimensionality and complexity of the topic.

## 4. Discussion

Upon analyzing the studies, it is evident that the majority of them lack the application of a theoretical model or concept. Only a few have employed a theoretical model, with the theory of planned behavior being the most commonly used [12,14,15]. Other theories, such as the risk calculus theory [16], threat appraisal theory [16], stream of sustainable development theory [17], and spatial and relational resilience theories [18], have also been used. However, it is apparent that there is a lack of theoretical foundation for the new approach aimed at measuring the impact of COVID-19 on the food retail sector. Therefore, future studies should investigate this area by using various conceptual theories to strengthen the theoretical basis of this topic. One possible theory that could be applied to the topic of the impact of COVID-19 on the food retail sector is the resource-based view (RBV) theory. This theory posits that a firm’s resources and capabilities are key determinants of its performance and competitive advantage [19]. Applying the RBV theory to the food retail sector in the context of COVID-19 could help identify how certain resources and capabilities, such as supply chain management, technology adoption, and workforce agility, can affect a firm’s ability to adapt and survive during a crisis. Additionally, the Technology Acceptance Model [20], can serve as a valuable tool for analyzing changes in consumer behaviors. This is particularly relevant in light of the current COVID-19 pandemic, which has prompted many companies to adopt new digital technologies and solutions to meet the challenges posed by the crisis. Applying this theory to the food retail sector could help identify how external pressures, such as government regulations, consumer demand, and media scrutiny, can shape the industry’s response to the pandemic. Overall, the application of these theoretical frameworks and others can provide a more robust and nuanced understanding of the impact of COVID-19 on the food retail sector and help inform policymakers and practitioners in shaping a more resilient and sustainable future for the industry.

### Pre, during, and Post COVID-19

After analyzing the bibliometric analyses of various studies, it is clear that the majority of them concentrate on the resilience retail strategy during the pandemic (Table 3). Conversely, few studies give attention to the pre- and/or post-pandemic periods. Most of the studies focusing on the pre-pandemic period also cover the during and/or post-pandemic periods [21,22,23,24], with a small number solely examining the pre-pandemic period [25,26]. These studies primarily concentrate on food consumption behaviors and waste. In contrast, post-pandemic-oriented studies primarily focus on changes in consumer behaviors after the COVID-19 outbreak. The current state of research indicates a notable gap in the literature regarding the pre- and post-pandemic periods. To address this gap, future studies must emphasize changes in consumer behaviors, decision-making, and habits that have emerged after the pandemic. By doing so, researchers can fill the knowledge gap that currently exists in this area of research and contribute to a better understanding of how the pandemic has changed consumer behaviors in the retail industry. This knowledge can be valuable for companies and policymakers who seek to develop effective strategies for the future.

Studies on the pandemic period are of great importance as they provide insight into how the pandemic has affected different aspects of society. The studies mentioned above highlight some key areas that have been the focus of research during the pandemic, including price changes [32,36,38,65], policy implications [40,48,49,55], food safety [17,30,43], and consumer behaviors [12,71,81]. For instance, the studies on price changes have examined the effects of the pandemic on commodity prices and how it has impacted different sectors of the economy. Meanwhile, studies on policy implications have looked at how governments have responded to the pandemic, the effectiveness of different policies, and their impact on society. Additionally, studies on food safety have examined the measures put in place to ensure that food remains safe and accessible during the pandemic, while studies on consumer behavior have explored the changes in consumer attitudes and habits. Despite the extensive research conducted during the pandemic, there is still a research gap in understanding the long-term effects of the pandemic on various sectors of the economy, including the retail sector. While some studies have looked at the post-pandemic period [5,82], there is a need for further research to explore the lasting changes in consumer behavior, decision-making, and habits that were brought about by the pandemic. Additionally, there is a need for research to investigate the potential long-term effects of policy interventions implemented during the pandemic on various sectors of the economy. Understanding these issues will be crucial in developing effective strategies to address the challenges faced by different sectors of the economy in the post-pandemic period.

## 5. Policy Implications

### Future Research Agenda: B2B vs. B2C

After a thorough analysis of the available literature, it has been brought to my attention that the research conducted on the business-to-consumer (B2C) sector has been exclusively focused on the pandemic period. This implies that there is a considerable research gap in terms of post-pandemic consumption and purchasing behavior. Such a gap is significant since it limits our understanding of the consumer’s behavior in the absence of the pandemic and the factors that may influence it. It is interesting to note that the two primary areas of research in the B2C context are purchasing behavior and food waste behavior, which are two opposing phenomena. On the one hand, purchasing behavior refers to the act of acquiring new goods and commodities, while on the other hand, food waste behavior emphasizes strategies aimed at minimizing daily consumption. Given the importance of these two factors in the consumer’s life, it is crucial to conduct further research to understand the relationship between them and how they influence post-pandemic purchasing behavior. In addition, it is important to consider the impact of external factors such as government policies, economic conditions, and technological advancements on the B2C sector. Studies on purchasing behavior have explored the adoption and usage of new technologies, particularly delivery services, that were necessitated by the pandemic [15,16,53,83,84,85], as well as identifying factors that have influenced changing consumer behavior [62,68,70,73,86,87]. In contrast, the pandemic has brought sustainability-related issues to the forefront, including food waste and security from a consumer perspective. Researchers have analyzed socio-demographic and psychographic characteristics that could potentially impact household food wastage [43,49,52]. Despite the considerable attention given to these topics, there are still several research areas that remain unexplored. To address this literature gap, we propose several research topics in Table 4. These topics aim to further examine the intersection between consumer behavior, sustainability, and new technologies, as well as the impact of the pandemic on these areas. By exploring these research questions, we can gain a better understanding of the challenges and opportunities presented by the changing consumer landscape and inform policies and interventions to promote sustainable and responsible consumption.

In the context of COVID-19, a crucial aspect of resilient retail strategy is how companies and policymakers respond to the challenges posed by the pandemic. Through an analysis of existing studies, two recurring areas have emerged that focus on the impact of COVID-19 on food supply chains and retailers’ strategies. With regard to supply chain disruptions caused by the pandemic, prior research has explored its effects on the food supply chain [11,41,49,58,64]. These studies have explored the various disruptions that the supply chain has experienced and their impact on the food system, with researchers striving to identify both short- and long-term solutions to mitigate disruptions and ensure the resilience and sustainability of food supply chains. Previous findings indicate that the COVID-19 pandemic has significantly impacted food supply chains across various regions, with disruptions in transportation, supply chain operations, and customer behavior [49,58]. Despite the difficulties faced, governmental efforts such as logistical assistance and phased purchasing have aided in the recovery of the food supply chain’s durability. Policymakers have the potential to enhance the strength and resilience of small-scale production systems through the implementation of policies and reforms, including contract farming, farmer-producer organizations, and social safety nets, as suggested by Cariappa et al. [49]. Additionally, these studies provide valuable insights for retail establishments, suppliers, and government agencies in optimizing the management of their supply chains amid the ongoing pandemic.

In addition, a highly researched area is how retailers have adapted their strategies during the pandemic [10,17,47,56,61,64,74,88,89]. These studies investigate the impact of supply chain disruptions and lockdown measures on supermarkets and other retailers. The goal of the research is to identify strategies that retailers and other organizations can implement to maintain their service levels and minimize transmission risks during the pandemic. Another area of research focuses specifically on how certain food items have been affected by the COVID-19 pandemic [38,65]. Overall, the research indicates that the pandemic has had a significant impact on both the food supply chain and the retail sector. Panic buying at the beginning of the lockdown caused temporary shortages due to disruptions in the supply chain [57]. Nevertheless, the food supply chain demonstrated resilience despite the challenges in demand management. The pandemic has also brought to light issues related to food availability and socio-economic problems resulting from income loss. However, there are concerns about the impact of control measures, such as increased plastic packaging, cleaning chemicals, waste, and sanitation on environmental sustainability [64]. These studies emphasize the importance of collaboration among SMEs, suppliers, customers, and other stakeholders to co-create mutually beneficial, commercially sustainable value.

During the pandemic, the agricultural sector faced challenges in sales and production due to physical distancing and other social restrictions [90]. Nevertheless, a closed-loop strategy that fostered cooperation between farmers, the industrial sector, and retailers proved successful in maintaining stable production. The pandemic underscores the importance of adapting and building resilience. The authors suggest that future research should explore forming alliances to develop local food as part of place-centered community resilience strategies, instead of just addressing single-issue topics. Future research could also reveal the degree to which the recent COVID-19 pandemic has changed the shopping behaviors of rural versus urban consumers toward healthier foods and/or sustainable and green-oriented consumption [91,92]. Technology and food shopping apps might help consumers to improve their online buying decisions [93], thus future research could also focus on analyzing food decision-making depending on the use of mobile apps.

The studies also identified limiting and driving factors in the transition from grocery to e-grocery during the pandemic. While there was a general increase in the grocery trade and disproportionate growth in online grocery sales, there was little shift from grocery to e-grocery. This finding highlights the need to reconsider the timing of windows of opportunity and the associated vulnerability of innovations that depend on them. However, most studies have focused on the pandemic period, neglecting its long-term effects. Additionally, prior research has not adequately analyzed the impact of COVID-19 on companies’ digital transition. Therefore, based on prior research, we have developed some research questions that could enhance the theoretical and managerial implications of a resilient retail strategy in future studies (Table 4).

## 6. Conclusions

The scope of this research was to identify shared areas and potential interdisciplinary research opportunities between COVID-19 and retail strategy, by empirically documenting the intellectual framework, volume, and trends of knowledge development. To achieve these goals, we conducted a bibliometric analysis of 69 articles published in Scopus-indexed journals. In this section of the paper, we present our findings, summarize the limitations of the study, and suggest avenues for future research.

A potential drawback of this research concerns the formation of the sample database. The search procedure utilized multiple collections of keywords to identify pertinent records, assuming that the words present in the title, author-assigned keywords, or abstract provided a comprehensive representation of the connection between COVID-19 and retail resilience strategies. However, there is a possibility that some valuable perspectives were not included in the sample, despite article-by-article reading. Future research could also strengthen the analysis of food retail resilience in urban areas versus rural areas, as consumer expectations, habits, and consumption patterns during the COVID-19 pandemic might have been slightly different. While urban consumers were faced with severe restrictions and had only limited access to physical shopping, rural consumers could rely to a certain extent on self-consumption. However, for both rural and urban consumers, the recent pandemic brought increased access to online shopping. Future studies could also investigate the extent to which online shopping has generated a significant shift in consumption patterns in rural versus urban areas.

Future research may employ additional search methods, such as snowballing or citation analysis, to broaden the scope of the sample and minimize the risk of missing important contributions. Furthermore, it is worth noting that the current study focused exclusively on the food retail sector. Therefore, in order to shed light on the enduring impact of COVID-19, future investigations could broaden the scope of analysis by incorporating and/or contrasting multiple industries. This would enable a more comprehensive understanding of the pandemic’s ramifications, both within and beyond the realm of food retail.

## Figures and Tables

**Table 1:** Research strategy using the SCOPUS database.

Steps	Process	Input	Output
1	Search strategy	Searched field: resilient retail strategies before, during, and after COVID-19	Boolean operators (‘OR’ and ‘AND’)
		Keywords:	
		‘retail resilience’ AND ‘food’	
		‘retail resilience strategy’ AND ‘food’	
		‘retail’ AND ‘COVID-19’ OR ‘coronavirus’ OR ‘SARS-CoV-2’ AND ‘food’	
		‘retail resilience’ AND ‘COVID-19’ OR ‘coronavirus’ OR ‘SARS-CoV-2’ AND ‘food’	
		‘retail resilience strategy’ AND ‘COVID-19’ OR ‘coronavirus’ OR ‘SARS-CoV-2’ AND ‘food’	
		‘resilience strategy’ AND ‘COVID-19’ OR ‘coronavirus’ OR ‘SARS-CoV-2’ AND ‘food’	
2	Search limitation	The data were selected for the time interval from 2019 to 2022. Two categories, Business and Management, were chosen.	The selected time period and applied categories have limited the number of studies for each search.
3	Results	The results were exported in ‘.csv’ format for later use in the VOS viewer. Subsequently, the results were searched in other databases, such as Google Scholar, and will be downloaded and imported into Mendeley for detailed analysis of the selected publications.	Six ‘.csv’ files were created from SCOPUS, containing important and necessary bibliographic data for analysis.
4	Data cleaning	The files were imported into VOS Viewer software 1.6.19 to clean and remove duplicate studies, as well as for the analysis of bibliographic data.	The software resulted in 69 publications for the final analysis in VOS Viewer 1.6.19.

**Table 2:** Journals in which reviewed articles were published.

Journal	Number of Articles
Sustainability (Switzerland)	10
Socio-Economic Planning Sciences	5
Food Security	3
Global Food Security	3
Food policy	2
Cogent Social Sciences	2
International Journal of Retail and Distribution Management	2
Other	43

**Table 3:** Categorization of studies based on the phase of the COVID-19 pandemic.

Phase of COVID-19	Authors
Pre-COVID-19	[21,22,23,24,25,26,27]
During COVID-19	[10,11,12,14,16,17,18,21,22,23,24,27,28,29,30,31,32,33,34,35,36,37,38,39,40,41,42,43,44,45,46,47,48,49,50,51,52,53,54,55,56,57,58,59,60,61,62,63,64,65,66,67,68,69,70,71,72,73,74,75,76,77]
Post-COVID-19	[14,24,39,66,75,78,79,80]

**Table 4:** Future research topics: B2B vs. B2C.

B2C	Purchase & consumption behavior	How has the pandemic influenced consumer food retail shopping habits in terms of frequency, mode of shopping, and preferred products purchased? In what ways can consumers be encouraged to transition from their old, unsustainable purchasing behavior to more sustainable and eco-friendly habits? Have consumer values related to food waste and safety changed as a result of the pandemic? If so, in what ways? What are the lingering effects of the pandemic on consumer purchasing and consumption habits in the post-pandemic era, and how can food retailers adjust to meet these evolving needs and preferences? What is the influence of urban versus rural shopping behavior in the post-pandemic context on online retailers? To what extent have lockdowns and sanitary restrictions affected the shopping patterns of urban versus rural consumers?
	Food waste & safety	What are the key factors that influence consumer confidence in the safety and quality of food retail products during the pandemic? What role do sustainability and social responsibility play in shaping consumer perceptions and preferences for food retail brands during the pandemic? What factors influence consumers’ decision-making in relation to reducing food waste in the post-pandemic era? To what extent do consumer perceptions of food safety influence their purchasing behavior in the post-pandemic era? What role do packaging and labeling play in reducing food waste and improving food safety in the post-pandemic era? How have consumers’ preferences for local and organic food changed during the pandemic, and what impact does this have on food waste and safety? Are there differences regarding consumer food waste behavior in rural versus urban areas?
B2B	Supply chains	How can food supply chains be made more resilient to future shocks and disruptions, such as pandemics, extreme weather events, and geopolitical conflicts? What are the long-term impacts of panic buying and stockpiling on the food supply chain, and how can these effects be mitigated or prevented? What are the most pressing threats to food security in the current and future landscape, and how can governments and other stakeholders work to address these issues sustainably and equitably?
	Retailers strategy	What are the most effective strategies for promoting sustainable practices in the food industry, particularly in light of the increased use of plastic and cleaning chemicals due to COVID-19? How can small and medium-sized businesses collaborate with suppliers, customers, and other stakeholders to create mutually beneficial and commercially sustainable value, particularly in the context of unpredictable and rapidly evolving retail environments? How can appointments and other preventive measures to manage pandemics affect the assortment competition of retailers, and what are the implications of this for consumers and the broader retail industry? What are the most effective adaptation strategies for food retail companies in the face of COVID-19 and other disruptions, and how can these strategies be scaled up and replicated across different regions and sectors? How can local food sectors form alliances and articulate themselves as part of place-centered community resilience strategies, particularly in areas where there is a lack of linking social capital? What are the driving and limiting factors behind the growth of online grocery trade, and how can retailers and policymakers encourage more consumers to transition to e-grocery?

## Data Availability

Data is contained within the article.
